# Metabolic and Fluid Biomarkers Support Microglia Activation in Amyotrophic Lateral Sclerosis

**DOI:** 10.1002/acn3.70521

**Published:** 2026-08-30

**Authors:** Matteo Zanovello, Francesca Serani, Alen Bebeti, Federica Paredi, Giulia Quadrio, Andrea Fortuna, Giacomo Maria Minicuci, Elena Pegoraro, Annachiara Cagnin, Diego Cecchin, Gianni Sorarù

**Affiliations:** ^1^ Neuromuscular Unit, Department of Neurosciences University Hospital of Padova Padova Italy; ^2^ Department of Neuromuscular Diseases UCL Queen Square Institute of Neurology, UCL London UK; ^3^ Nuclear Medicine Unit, Department of Medicine University Hospital of Padova Padova Italy; ^4^ Padova Neuroscience Center University of Padova Padova Italy

**Keywords:** ALS, chitinase, CSF, microglia, PET‐MR

## Abstract

Amyotrophic lateral sclerosis is an incurable neurodegenerative disease involving motor neuron degeneration and metabolic and immune dysfunction. We combined clinical data, cerebrospinal fluid biomarkers and fluorodeoxyglucose positron emission tomography with magnetic resonance imaging to investigate the role of reactive microglia in disease pathogenesis. Patients showed increased cerebrospinal fluid levels of neurofilament light chain and chitinases, along with hypermetabolism in the medulla oblongata. Chitinase levels correlated with brainstem metabolism and clinical severity. These findings suggest a link between microglial activation, brainstem hypermetabolism and disease progression in amyotrophic lateral sclerosis, supporting a central role for neuroinflammation in disease pathogenesis.

## 1 Introduction

Amyotrophic lateral sclerosis (ALS) is an incurable neurodegenerative disorder characterised by the progressive loss of motor neurons leading to death [1]. Despite supportive contributions from neuroimaging [2] and cerebrospinal fluid (CSF) analysis [3], the diagnosis of ALS is primarily based on clinical examination and electrophysiological tests.

While the aetiology of ALS remains incompletely understood, the disease pathogenesis has traditionally been attributed to cell‐autonomous mechanisms within motor neurons [1]. However, non‐cell‐autonomous mechanisms, particularly neuroinflammation, have emerged as key pathological features, with major contributions from astrocytes and microglia. Previous studies have shown alterations in astrocyte‐mediated glutamate reuptake and metabolic support to neurons. In parallel, microglia have been reported to shift from a neuroprotective to a neurotoxic phenotype [4].

In line with this, CSF biomarkers provide a complementary window into ALS pathophysiology, with elevated neurofilament light chain (NfL) levels reflecting neuronal injury [3]. Additionally, increased concentrations of chitinases, a family of enzymes involved in immune system regulation and inflammation, have been detected in the CSF of ALS patients and are associated with disease severity and progression [5, 6]. Chitinases include chitotriosidase (CHIT1), chitinase‐3‐like protein 1 (CHI3L1) and chitinase‐3‐like protein 2 (CHI3L2), which are secreted by reactive glial cells in the central nervous system (CNS) [7]. CHIT1 and CHI3L2 are predominantly expressed by microglia, although CHI3L2 has also been detected in astrocytes. In contrast, CHI3L1 is mainly expressed by activated astrocytes [5, 8, 9].

Neuroimaging studies using ^18^F‐fluorodeoxyglucose positron emission tomography ([^18^F]FDG PET) have identified metabolic alterations in various brain regions in ALS [10, 11], including increased [^18^F]FDG uptake in the brainstem, possibly reflecting glial activation [11]. However, the cellular basis and clinical significance of these changes remain unclear, and only a few studies have integrated nuclear medicine and CSF data from the same individuals to explore the relationship between metabolic activity and neuroinflammation [12, 13].

In this study, we assess brainstem metabolism and CSF levels of microglial‐associated markers in ALS patients and controls, as well as associate them with clinical variables and patient survival.

## 2 Methods

Twenty‐five patients (11 males and 14 females) with a clinical diagnosis of ALS were enrolled; 5 of them had bulbar onset, and 20 had spinal onset. ALS Functional Rating Scale–Revised (ALS‐FRS‐r) scores were collected at diagnosis. Disease progression rate (ΔALS‐FRS‐r) was calculated as (48 − ALS‐FRS‐r at diagnosis) divided by the time from symptom onset to diagnosis (months). Two ALS patients lacking ALS‐FRS‐r data and three lacking survival data (non‐overlapping) were excluded, yielding *n* = 23 for functional and *n* = 22 for survival analyses. PET/MR controls (*n* = 19) were clinical controls from the Nuclear Medicine Unit who underwent the same imaging protocol for non‐neurological/non‐CNS indications; detailed individual histories were unavailable due to anonymisation. CSF controls (*n* = 10) had no clinical or neuroradiological evidence of neurological disease (Table 1). The study was approved by the local ethics committee (protocol AOP1673‐4831/AO/20). All participants provided written informed consent in accordance with the Declaration of Helsinki, and data were anonymised before analysis.

**TABLE 1:** Demographics and clinical information for the study cohorts.

	ALS	CSF controls	PET controls
Sex	Males 11, females 14	Males 2, females 8 (Fisher exact test, *p* = 0.071)	Males 13, females 6 (Fisher exact test, *p* = 0.535)
Site of onset	Spinal 20, bulbar 5	—	—
Age at onset (years)	58 (43–79)	—	—
Age at evaluation (years)	60 (43–83)	53.3 (20–73) (Wilcoxon test, *p* = 0.016)	49 (19–81) (Wilcoxon test, *p* = 0.016)
Survival (months)	50 (20–132)	—	—
Inflammatory/autoimmune comorbidities	Psoriasis (2), rheumatoid arthritis (1), autoimmune thrombocytopenia (1), chronic inflammatory demyelinating polyneuropathy (1), post‐streptococcal glomerulonephritis (1), polymyalgia rheumatica (1)	Rheumatoid arthritis (1)	—

*Note:* Age and survival are expressed as median and range.

According to European Association of Nuclear Medicine guidelines [14], participants fasted for at least 6 h before the injection of the radiopharmaceutical, and blood glucose levels were required to be less than 200 mg/dL. A 3 MBq/kg intravenous injection of [^18^F]FDG was administered under resting conditions 60 min before scanning. Simultaneous PET/MR acquisition was performed using a Siemens Healthcare Biograph mMR system, including T1‐weighted (1 mm isotropic) MRI and PET brain imaging. Image post‐processing was conducted via PMOD software. Volumes of interest (VOIs) were obtained through PMOD automated segmentation and parcellation on combined PET/MR data. All segmentations were visually inspected and manually corrected where necessary. An Automated Anatomical Labelling atlas was used for spatial normalisation and to define specific VOIs—notably the midbrain, pons, medulla oblongata and parieto‐occipital white matter. Mean uptake values were normalised to occipital white matter, selected as reference due to its relative metabolic preservation [11].

CSF concentrations of CHIT1 and CHI3L2 were measured using commercially available sandwich ELISA kits (Circulex and MBL Research Products, respectively). NfL levels were quantified using the UmanDiagnostics NF‐light ELISA. All samples and standards were analysed in duplicate according to the manufacturer's instructions.

Group comparisons were performed using the Wilcoxon rank‐sum test and linear regression with age as a covariate to address the lack of age matching between groups. Associations between continuous variables were assessed using Spearman correlation, and significant associations were further tested by linear regression with age as a covariate and multiple testing correction (Benjamini–Hochberg). Survival analyses were conducted using the log‐rank test and the Cox proportional hazards regression model with age at diagnosis as a covariate. Differences in [^18^F]FDG uptake and CSF biomarker levels between bulbar‐ and spinal‐onset patients, and between CSF biomarkers and autoimmune/inflammatory comorbidities, were assessed using the Wilcoxon test. Statistical significance was set at *p* < 0.05.

## 3 Results

**FIGURE 1:**
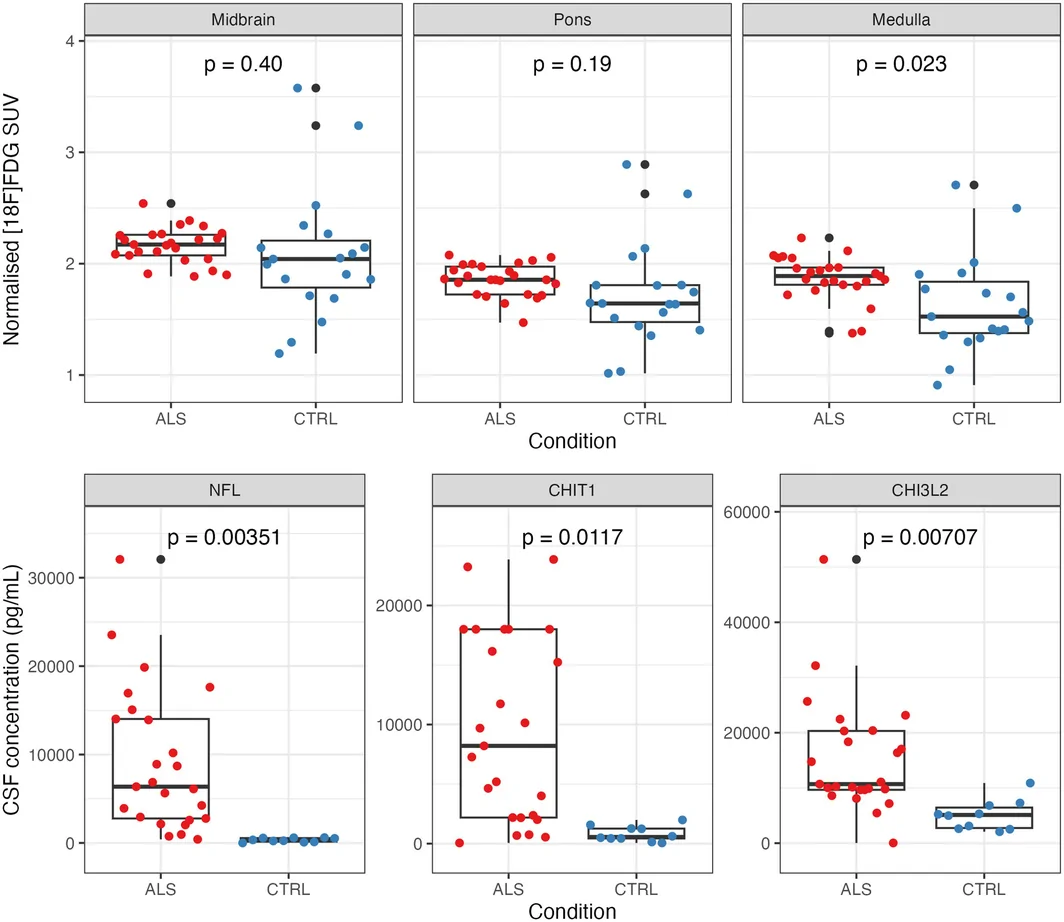
(Top) Normalised [18F]FDG uptake values in the midbrain (left), pons (middle) and medulla oblongata (right) for ALS patients (*n* = 25) and controls (*n* = 19). (Bottom) CSF concentrations of NfL (left), CHIT1 (middle) and CHI3L2 (right) for ALS patients (*n* = 25) and controls (*n* = 10). *p* values are derived from linear regression models adjusted for age.

First, we evaluated metabolic patterns in the midbrain, pons and medulla oblongata in 25 ALS patients and 19 controls using [^18^F]FDG PET/MRI [11]. We observed a statistically significant difference between ALS patients and controls in the medulla oblongata (Figure 1, top; linear model corrected for age, *p* = 0.023). Next, we assessed CSF concentrations of NfL, CHIT1 and CHI3L2 in the same ALS cases and in 10 controls. All three biomarkers were significantly elevated in ALS compared with controls (Figure 1, bottom; linear model corrected for age, NfL *p* = 0.00351, CHIT1 *p* = 0.0117, CHI3L2 *p* = 0.00707) [6]. No significant differences were observed in brainstem [^18^F]FDG uptake or CSF biomarkers between bulbar and spinal onset ALS. Peripheral inflammatory indices—neutrophil‐to‐lymphocyte ratio, platelet‐to‐lymphocyte ratio, systemic immune‐inflammation index and lymphocyte‐to‐monocyte ratio—did not correlate with CSF biomarkers, and autoimmune/inflammatory comorbidities (Table 1) had no impact on CHIT1, CHI3L2, or NfL levels.

Correlation analysis revealed significant correlations between the two chitinases (*r* = 0.7, *p* < 0.0001) and among [^18^F]FDG SUV in the three brainstem regions (midbrain‐pons *r* = 0.86, *p* < 0.0001; midbrain‐medulla *r* = 0.76, *p* < 0.0001; pons‐medulla *r* = 0.82, *p* < 0.0001) within the ALS group. CHI3L2 levels positively correlated with [^18^F]FDG uptake in the medulla oblongata (*r* = 0.42, *p* = 0.0364), while CHIT1 concentrations correlated with glucose uptake in both the medulla oblongata (*r* = 0.41, *p* = 0.0426) and the pons (*r* = 0.42, *p* = 0.0343). Additionally, CSF NfL levels correlated with both chitinases (CHIT1 *r* = 0.67, *p* < 0.0001; CHI3L2 *r* = 0.84, *p* < 0.0001) and with glucose uptake in the medulla oblongata (*r* = 0.48, *p* = 0.0169). We also examined associations with clinical measures, observing negative correlations of the ALS‐FRS‐r with NfL (*r* = −0.52, *p* = 0.0104), pons and medulla oblongata [^18^F]FDG uptake (r = −0.54, *p* = 0.00812; and *r* = −0.45, *p* = 0.0315, respectively) and CHI3L2 (r = −0.62, *p* = 0.00162), and positive correlation of the ΔALS‐FRS‐r with NfL (*r* = 0.76, *p* < 0.0001), medulla oblongata [^18^F]FDG uptake (*r* = 0.48, *p* = 0.0197) and CHI3L2 (*r* = 0.64, *p* = 0.0081). Multivariate regression analyses, adjusted for age and corrected for multiple testing, showed that higher medulla oblongata metabolism was independently associated with higher CSF CHIT1 (β = 0.473, *p*_adj = 0.028), CHI3L2 (β = 0.470, *p*_adj = 0.028) and NfL levels (β = 0.468, *p*_adj = 0.028), as well as lower ALS‐FRS‐r scores (β = −0.542, *p*_adj = 0.028). Higher pontine metabolism was independently associated with higher CHIT1 levels (β = 0.500, *p*_adj = 0.022). CSF biomarkers were strongly interrelated, with positive associations between CHIT1 and CHI3L2 (β = 0.571, *p*_adj < 0.001), CHIT1 and NfL (β = 0.499, *p*_adj = 0.003), and CHI3L2 and NfL (β = 0.761, *p*_adj < 0.001). Higher CHI3L2 and NfL levels were independently associated with lower ALS‐FRS‐r scores (β = −0.732, *p*_adj = 0.003 and β = −0.705, *p*_adj = 0.004, respectively).

**FIGURE 2:**
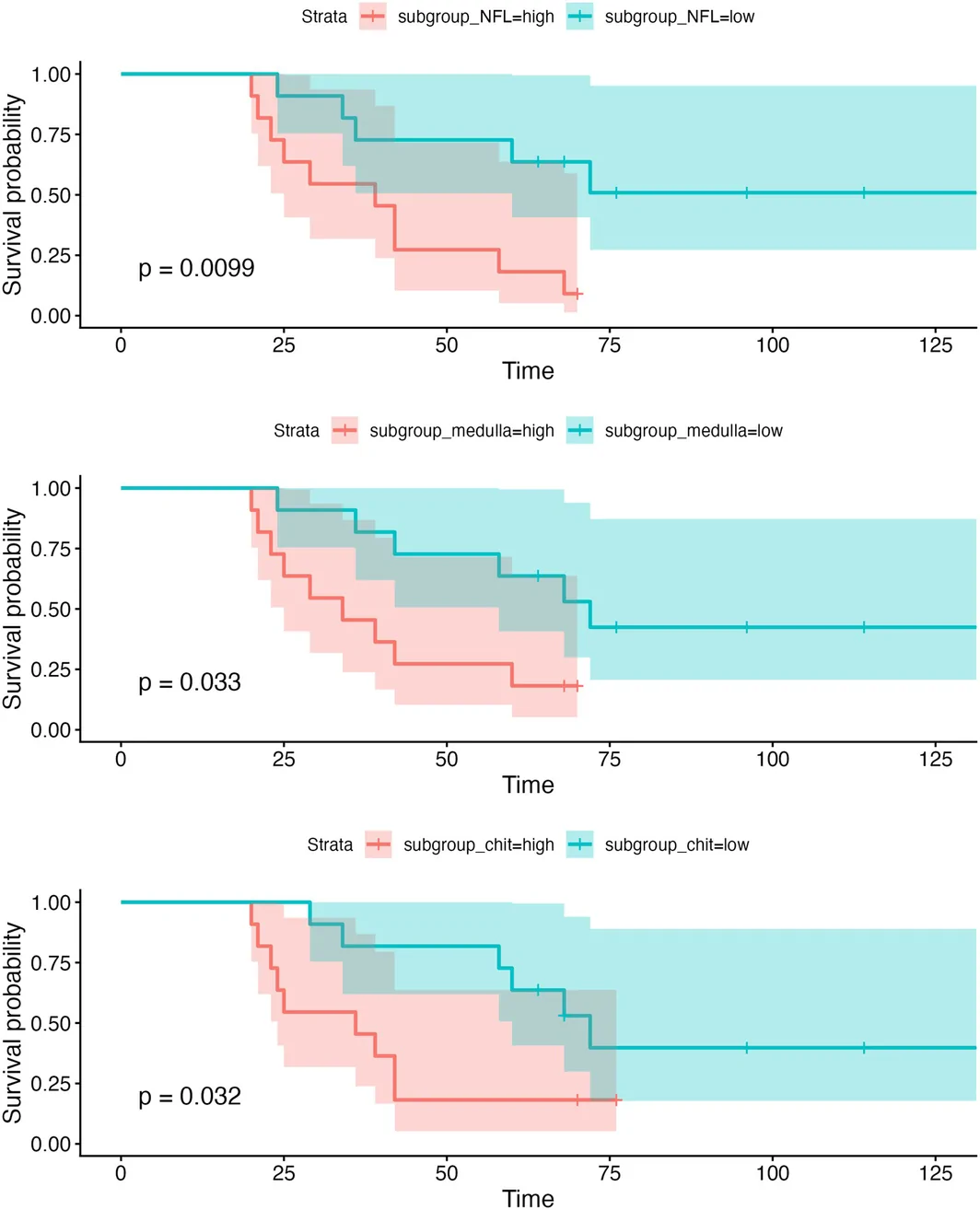
(Top) The 11 patients with CSF NfL over 50th percentile showed significantly shorter survival than the 11 patients with CSF NfL below 50th percentile. (Middle) The 11 patients with medulla oblongata uptake over 50th percentile showed significantly shorter survival than the 11 patients with medulla oblongata uptake below 50th percentile. (Bottom) The 11 patients with CSF CHI3L2 over 50th percentile showed significantly shorter survival than the 11 patients with CSF CHI3L2 below 50th percentile. Survival is expressed in months from the symptom onset. *p* value was calculated through log‐rank test.

We retrospectively evaluated the association of baseline NfL concentrations, medulla oblongata glucose metabolism, and CHI3L2 levels and ALS survival by dichotomising the cohort according to the median. Patients with higher values of each marker exhibited shorter survival compared with those with lower levels (Figure 2, log‐rank test: NfL *p* = 0.0099, medulla oblongata [^18^F]FDG uptake *p* = 0.033, CHI3L2 *p* = 0.032). We confirmed the finding by Cox proportional hazards regression model with age at diagnosis as a covariate (NfL *p* = 0.00044, medulla oblongata [18F]FDG uptake *p* = 0.047, CHI3L2 *p* = 0.0015).

## 4 Discussion

In this study, we found that ALS patients exhibited elevated [^18^F]FDG uptake in the medulla oblongata and increased CSF concentrations of CHIT1 and CHI3L2. These measures were significantly correlated with each other and were associated with NfL levels and clinical outcomes, including survival. The correlations between chitinases and brainstem metabolism remained significant after age adjustment and correction for multiple testing, supporting the robustness of these associations within the constraints of the current sample size. Our findings support a model in which localised metabolic changes in the brainstem reflect underlying microglial activation, with CHI3L2 emerging as a potential prognostic biomarker.

Neuroinflammation has been increasingly recognised as a hallmark of ALS pathophysiology [4]. Our findings extend prior work by showing that elevated brainstem metabolism, particularly in the medulla oblongata, may reflect an active neuroinflammatory process rather than neurodegeneration alone [11]. Although the controls were younger than the patients, they exhibited lower brainstem metabolism, contrary to what would be expected; this observation supports the reliability of the results. Moreover, this interpretation is supported by the concurrent increase in CSF microglial markers, notably CHIT1 and CHI3L2, both of which are considered indicators of microglial activation [6, 15]. While previous PET studies have shown metabolic alterations in ALS, only a few have directly linked these metabolic changes with CSF biomarkers in the same cohort, focusing on NfL only [12, 13]. Our findings of brainstem hypermetabolism alongside elevated CSF chitinases align with prior PET evidence of microglial (TSPO) and astrocytic (MAO‐B) activation in motor regions of ALS patients [16]. While [^18^F]FDG cannot distinguish glial subtypes, the spatial and biochemical concordance supports a neuroinflammatory glial contribution to the observed hypermetabolism. Combined [^18^F]FDG/TSPO or MAO‐B PET in future cohorts could clarify relative microglial versus astrocytic contributions.

No significant differences in brainstem metabolism or CSF biomarkers were found between bulbar‐ and spinal‐onset patients, although the small bulbar subgroup warrants caution. CSF markers did not correlate with peripheral inflammatory indices and were not affected by autoimmune/inflammatory comorbidities, supporting a primarily CNS‐compartmentalised glial origin rather than systemic immune activation.

Elevated CHI3L2 levels were associated with poorer survival outcomes, supporting prior reports implicating this protein in neuroinflammatory cascades and disease progression in ALS [6], and suggesting a role for CHI3L2 as a prognostic indicator.

Our study also highlights the utility of multimodal biomarker approaches in ALS. The significant correlations observed between imaging‐derived metabolic activity and CSF microglial markers, as well as their relationship with survival, suggest that these tools may reflect overlapping pathophysiological processes. Integrating these markers could enhance prognostication and stratification into subgroups with distinct clinical trajectories, offering potential for more personalised disease monitoring in ALS clinical trials.

This study has several limitations. The PET/MR and CSF control groups differed in composition and were not age‐matched to the ALS cohort, though age was controlled for statistically; neither group consisted of healthy volunteers, and detailed PET/MR control histories were unavailable due to anonymisation. The modest sample size limits statistical power and generalisability. Additionally, although we focused on brainstem regions, future studies should investigate other brain regions and the spinal cord, previously shown to be altered in ALS [10, 17], to provide a more comprehensive understanding. Longitudinal studies focusing on larger, multicentre cohorts will also be necessary to assess the dynamics of these biomarkers over time. Moreover, it will be essential to include blood samples to evaluate the relationship between serum and CSF chitinase levels [18, 19], and postmortem tissue to elucidate the pathological expression of these proteins [20]. Finally, these findings should be interpreted in light of patients' genetic status, particularly given the presence of a CHIT1 polymorphism linked to ALS [21].

In conclusion, we demonstrated increased metabolic activity in the medulla oblongata, along with elevated CSF microglial markers, particularly CHI3L2. Taken together, these findings further suggest a role for activated microglia in the pathogenesis of ALS. These biomarkers show promise for improving prognosis and patient stratification in both research and clinical contexts.

## Author Contributions

**Matteo Zanovello, Diego Cecchin, Gianni Sorarù:** conceptualisation. **Matteo Zanovello, Annachiara Cagnin, Diego Cecchin, Gianni Sorarù:** methodology. **Matteo Zanovello:** software. **Matteo Zanovello:** formal analysis. **Matteo Zanovello, Francesca Serani, Alen Bebeti, Federica Paredi, Giulia Quadrio, Andrea Fortuna, Giacomo Maria Minicuci, Elena Pegoraro, Diego Cecchin, Gianni Sorarù:** data curation. **Matteo Zanovello:** writing – original draft preparation. **Matteo Zanovello, Annachiara Cagnin, Diego Cecchin, Gianni Sorarù:** writing – review and editing. **Diego Cecchin, Gianni Sorarù:** project administration. All authors read and agreed to the published version of the manuscript.

## Funding

The authors have nothing to report.

## Conflicts of Interest

The authors declare no conflicts of interest.

## Data Availability

Data are available upon reasonable request to the corresponding author.
